# The advent of Onco-nephrology – a novel subspecialty

**DOI:** 10.12861/jrip.2014.19

**Published:** 2014-06-11

**Authors:** Mohammed-Mahdi Althaf

**Affiliations:** Department of Medicine, Section of Nephrology, King Faisal Specialist Hospital and Research Center, Riyadh, Kingdom of Saudi Arabia

**Keywords:** Onco-nephrology, Acute Kidney Injury, Chemotherapy, Cancer

**Implication for health policy/practice/research/medical education:**

The term onco-nephrology has been coined in an attempt to highlight the evolution of an increasingly complex and important sub-specialty in the practice of renal medicine.

The term onco-nephrology has been coined in an attempt to highlight the evolution of an increasingly complex and important sub-specialty in the practice of renal medicine. Given the improved survival and aging population, the prevalence of comorbidities is ever expanding and chronic kidney disease (CKD) is a major constituent. Between 2005 and 2009 >75% of cancer diagnoses occurred in individuals aged ≥65 years (1). When the words ‘Kidney’ and ‘Cancer’ are read in the same sentence several links between the two prop up in the mind of a nephrologist. It is important to note in cancer patients that the prevalence, cause and rate of progression of acute kidney injury (AKI) as well as CKD are different when compared to the general population. The one-year risk of AKI (defined as a >50% rise in serum creatinine) in patients with cancer was 17.5%, with a 27% risk over 5 years (2). Nephrologists are involved in several levels of care for patients with cancer. In end-stage renal disease (ESRD) patients on hemodialysis or peritoneal dialysis we screen patients for certain common malignancies in addition to age appropriate screening. Dialysis patients are more prone to develop bladder cancer, renal cell carcinoma, hepatocellular carcinoma, thyroid cancers, tongue cancers, cancer of the cervix, multiple myeloma and non-Hodgkin lymphoma. However, the incidence of other solid tumors are not more common than the general population (3,4). We have previously described a system that we employed where patients in a dialysis unit are screened for malignancies as well as other chronic ailments in a setting where a general practitioner is not involved (5). During pre-transplant recipient work-up we screen for malignancies and also decide on when a patient is fit for transplantation after a period of remission from a cancer. With renal transplant recipients we balance immunosuppression and allograft function whilst monitoring them vigilantly for malignancy. In patents with active malignancy who undergo several contrast enhanced imaging studies; we deal with contrast nephropathy risk assessment as well as management if AKI does occur. Furthermore, we deal with complex scenarios of electrolyte and fluid disturbances that are often encountered with an oncology patient. The most common paraneoplastic syndrome being AKI resulting from volume depletion related to malignancy treatment-associated malnutrition (6). Other settings include management of tumor lysis syndrome, thrombotic microangiopathy, obstructive uropathy, cancer associated hypercalcemia, lymphomatous infiltration of the kidney, kidney disease in hematopoietic cell transplantation, hepatic veno-occlusive disease and hypertension induced by anti-angiogenic therapies. Interestingly there are renal manifestations of several malignancies, namely- membranous nephropathy, cast nephropathy and monoclonal immunoglobulin deposition disease and several others. The burst of newer targeted chemotherapy over the past two decades has resulted in improved outcomes for several types of cancer. These are often used in different combinations at high doses that are potentially nephrotoxic; each with a unique pattern of toxicity leaving the nephrologist puzzled with which offender agent was responsible. The renal injury may manifest as AKI, a specific electrolyte imbalance, progressive CKD or a combination of the three. Table 1  adapted from an article published by Perazella (7) illustrates the type of kidney injury classified by the different compartments of the kidney that are associated with chemotherapy. Patients with malignancy now have greater survivorship and as a result are being administered aggressive sequential chemotherapy regimens with a protracted course—further contributing to nephron loss. Another area where our expertise is often sought for is in dosing of chemotherapy agents in the setting of CKD. The decision to withhold, initiate or discontinue renal replacement therapy when it comes to ESRD or AKI is not simple. It should be done after careful assessment of the patent’s status, expected clinical outcome as well as life expectancy in a collaborative meeting with the patient or patient’s family, oncologist, nephrologist and any other specialties involved in the overall care of the patient. It is evident that the discipline of ‘Onco-nephrology’ has inevitably evolved on basis of need. This article highlights the different subpopulations of patients encountered in the practice of nephrology where adequate knowledge of oncology is mandated. Over the last two years Onco-nephrology has gained much attention in several nephrology meetings and publications in an attempt to increase awareness in the nephrology community. With the rapidly progressing field of oncology and chemotherapeutic regimes, it is our responsibility as nephrologists to keep at pace and adapt to the changes that lie ahead so that we can provide optimal care for oncology patients with renal problems.

**Table 1:** Chemotherapy associated kidney injury classified by renal compartments.

**Compartment**	**Kidney Injury**	**Agent**
**Renal Vasculature**	Hemodynamic AKI (capillary leak syndrome)	• IL-2 • Denileukin diftitox
	Thrombotic microangiopathy (TMA)	• Antiangiogenesis drugs (bevacizumab and tyrosine kinase inhibitors) • Gemcitabine and Cisplatin • Mitomycin C and IFN
**Glomeruli**	Minimal change disease (MCD)	• IFN • Pamidronate
	Focal segmental glomerulosclerosis (FSGS)	• IFN • Pamidronate • Zoledronate (rare)
**Tubulointerstitium**	Acute tubular necrosis (ATN)	• Platinums • Zoledronate • Ifosfamide • Mithramycin • Pentostatin • Imatinib • Diaziquone • Pemetrexed
	Fanconi syndrome	• Cisplatin • Ifosfamide • Azacitadine • Diaziquone • Imatinib • Pemetrexed
	Salt wasting	• Cisplatin • Azacitidine
	Hypomagnesemia	• Cisplatin • Cetuximab • Panitumumab
	Nephrogenic diabetes insipidus (DI)	• Cisplatin • Ifosfamide • Pemetrexed
	Syndrome of inappropriate antidiuretic hormone secretion (SIADH)	• Cyclophosphamide • Vincristine
	Acute interstitial nephritis	• Sorafenib • Sunitinib
	Crystal nephropathy	• Methotrexate

## Author’s contribution

MMA is the single author of the manuscript.

## Conflict of interests

The author declared no competing interests.

## Ethical considerations

Ethical issues (including plagiarism, data fabrication, double publication) have been completely observed by the author.

## Funding/Support

None.
